# Tibial Derotational Osteotomy for Patellofemoral Instability: A Systematic Review

**DOI:** 10.1155/2022/8672113

**Published:** 2022-12-28

**Authors:** Phillip Wyatt, James Satalich, Zylyftar Gorica, Conor O'Neill, John Cyrus, Alexander Vap, Robert O'Connell

**Affiliations:** Virginia Commonwealth University School of Medicine, Richmond, VA, USA

## Abstract

**Introduction:**

The etiology of patellofemoral (PF) instability is multifactorial. Excessive external tibial torsion has been associated with recurrent patellar subluxation and persistent anterior knee pain. Several surgical techniques have been historically used to correct this, including medial patellofemoral ligament reconstruction, tibial tuberosity transfer (TTT), trochleoplasty, and tibial derotation osteotomy (TDO). The purpose of this systematic review is to investigate the safety and efficacy of TDO for PF instability and pain.

**Methods:**

A thorough search of the literature was conducted on July 15, 2022. Seven studies met the inclusion criteria for this systematic review.

**Results:**

Among the included studies, there were 179 total subjects and 204 operative knees. Mean follow-up time was 66.31 months (range 11–192). Complication rate was low (12.8%) in studies that reported complications. Average degree of anatomical correction in the transverse plane was 19.9 degrees with TDO. This increased to 34 degrees when combined with TTT. All PROMs assessed were significantly increased postoperatively (*p* < 0.05). Age greater than 25 years and advanced PF chondromalacia may negatively affect postoperative outcomes.

**Conclusion:**

The primary findings of this review were as follows: (1) TDO results in significantly improved pain and PROM ratings in patients with PF pain and/or instability, (2) the likelihood of complication, including recurrent patella subluxation after TDO, is low but may be increased by aging, and (3) the successful anatomical correction of TDO may be augmented by concurrent TTT in some cases.

## 1. Introduction

Patellofemoral (PF) instability affects 23 in 100,000 person-years, with the majority of these cases occurring in young females [1]. Patellofemoral pain and instability can be caused by a variety of underlying conditions, including but not limited to insufficient medial patellofemoral ligament, patella alta, alignment deformity in the coronal plane, dysplastic trochlea, femoral torsional malalignment, or increased tibial tuberosity-trochlear groove distance (TTTG) [2–7]. Tibial torsion deformities have also been associated with patellofemoral (PF) pain and instability in the literature [8, 9]. Malignment of the patella on the femur, creating abnormal mechanics at the patellofemoral joint (PFJ), appears to play a significant role in the development of PF pain and instability by causing undue stress on the ligamentous and cartilaginous components of the joint. Malignment, especially within the transverse and coronal planes, can also predispose patients to recurrent subluxation of the joint, a common clinical presentation in young athletes, particularly females [10–12]. However, the etiology of PF instability and pain is multifactorial, making it difficult to choose the appropriate surgical technique for patients with PF pathology [13].

Excessive external tibial torsion (EETT) is considered more than 30 degrees about the tibial shaft within the transverse plane as observed on radiographic imaging [14]. However, EETT in the absence of PF instability or pain is not considered an absolute indication for surgical correction [15]. Historically, tibial tuberosity transfer (TTT) has been used to realign the knee extensor mechanism in symptomatic PF instability. However, it has been shown that in patients with EETT, a TTT alone may increase medial tibiofemoral contact pressure and change tibiofemoral joint loading, leading to PF osteoarthritis [16]. For this reason, tibial derotation osteotomy (TDO) has increased in popularity among surgeons who treat PF instability and pain. A TDO is often performed in combination with or in lieu of a TTT [17–19].

The purpose of this systematic review is to synthesize salient findings from the current body of literature on TDO for PF instability and pain. More specifically, this systematic review will investigate the safety and efficacy of TDOs as determined by postoperative anatomical measurements, patient-reported outcome measures (PROMs), and postoperative complications in order to better understand the utility of this promising surgical technique.

## 2. Methods

This is a systematic review of studies on the short- and long-term outcomes associated with tibial derotation osteotomy procedures indicated for patellofemoral pathology. The search was conducted on July 15, 2022. The PRISMA (preferred reporting items for systematic reviews and meta-analyses) guidelines were used to report the process of article selection [20]. These guidelines have been used in prior similarly designed studies [21].

### 2.1. Search Strategy

One author (J.C.) searched the Pubmed/Medline, Cochrane, CINAHL, and Embase (OVID) databases on July 15, 2022. This search was repeated by one other author (P.W.) and verified by a third (J.S.). Combinations of keywords and controlled vocabulary were used to search for the concepts of rotation/derotation, tibia, and torsion (Table 1).

### 2.2. Screening, Inclusion/Exclusion Criteria

One author (P.W.) independently screened all the articles using the following inclusion criteria: studies that were published in 2010 and later, studies that included human subjects who underwent a tibial derotation osteotomy as indicated for patellofemoral pathology, and studies that assessed outcomes longer than 1 year postoperatively. Exclusion criteria were as follows: studies published prior to 2010, studies without a full-text version written in the English language, and studies that did not assess outcomes beyond 1 year postoperatively. Case reports were excluded from this study.

### 2.3. Data Extraction

**Figure 1:**
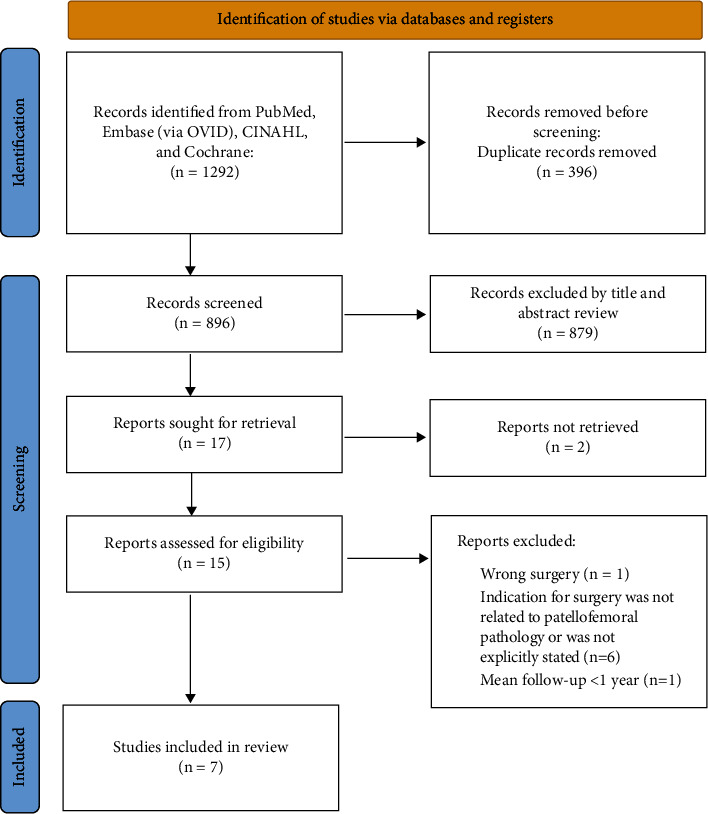
PRISMA 2020 flow diagram depicting the exclusion/inclusion process.

Data were extracted systematically for the aim, sample size, relevant methodological design, outcomes, findings, and adverse events. The initial search returned 1292 articles; 396 duplicates were removed, leaving 896 articles to screen. Titles and abstracts of these articles were screened initially for inclusion/exclusion criteria. This left 17 articles for full-text screening. Two were excluded due to no full-text version being readily available [22, 23]. One was excluded because the subjects did not have a surgery that included a tibial derotational osteotomy [24]. One was excluded due to follow-up time being less than 1 year postoperatively [3]. Six more were excluded because the indication for surgery did not include patellofemoral pathology [5, 8, 25–28]. This left 7 articles that met the inclusion criteria [17–19, 29–32]. The inclusion/exclusion process is depicted in Figure 1.

### 2.4. Quality Assessment

Two authors (P.W. and J.S.) assessed the risk of bias for all included articles using the Quality Assessment Tool for Case Series Studies, published by the National Institutes of Health (NIH) [33]. Six of the studies were determined to have a low risk of bias meeting at least 7 of the 9 items listed on the assessment tool [17–19, 30–32]. Three studies met all 9 criteria [19, 30, 32]. Three studies met 8 out of 9 criteria [14, 15, 17]. One study met 7 out of 9 criteria [29]. Based on this assessment, the risk of bias for the seven included articles was determined to be low.

## 3. Results

### 3.1. Characteristics of Subjects

All seven included studies were case series, one of which was prospective [32]. The cumulative number of subjects was 178 (34 males, 144 females) with a total of 204 operative knees that underwent a tibial rotational osteotomy. The mean age of the subjects was 27.83 years old (range 13–62). The mean follow-up time for the remaining five studies was 66.31 months (range 11–192). Salient findings from each included study can be found in Table 2. Information regarding the average anatomical correction among the studies is given in Table 3. Many of the studies assessed patient-reported outcome measures, including the Knee Society Score, Kujala Patellofemoral Score, Short-Form-12, and the Visual Analogue Scale. All studies reported significant improvements according to these outcome measures. These outcomes are described in Table 4.

### 3.2. Postoperative Complications

One study with 20 TDOs reported no postoperative complications [29]. The average complication rate for the remaining 6 studies was found to be 12.8% (range 8.3%–50%) [17–19, 30–32]. The most common complication observed was the presence of arthrofibrotic adhesions, requiring subsequent open surgical release (*n* = 6) [14, 16, 25–27] or manipulation under anesthesia (*n* = 2) [19]. Transient common fibular nerve neuropraxia was encountered in four subjects, which all demonstrated spontaneous resolution of neuropraxia [19, 30–32]. Other complications included persistent anterior knee pain (*n* = 2) [17], nonunion of the tibia (*n* = 3) [18, 32], permanent palsy of extensor hallucis longus muscle (*n* = 1) [30], compartment syndrome (*n* = 1) [32], and persistent anterior knee pain (*n* = 2) [17]. One case of persistent anterior knee pain required subsequent patellar resurfacing [17]. Additionally, 13 subjects (7.26%) were reported to require hardware removal due to postoperative pain [17, 30]. Details regarding complications reported in each study are outlined in Table 2.

### 3.3. Miscellaneous Findings

Several subjective questionnaires were given to patients at follow-up, providing valuable insight into patients' perceptions of the outcome of their operation. Two studies asked patients at follow-up whether they would undergo the procedure again. One study reported that 80% would undergo the procedure again (*n* = 10, mean follow-up = 81 months) [17]. The other reported that 91.8% would undergo the procedure again (*n* = 49, mean follow-up = 42 months) [32]. Stevens et al. found that 56% of subjects reported “trusting” their knee at a mean follow-up time of 85 months [31]. Again, this finding may be confounded by the inclusion of surgical techniques other than TDO.

In their statistical analysis, Manilov et al. assessed six potential factors that may influence poor outcomes after TDO using the Kujala PROM: (1) presence of femoral anteversion preoperatively (>20 degrees), (2) chondromalacia grade, (3) age group, (4) concomitant lateral retinacular release, (5) body mass index (BMI), and (6) history of previous surgery on the operative leg [30]. They found that advanced chondromalacia (grades 2–4), age 25 years or older, and history of prior knee surgery portended a worse postoperative Kujala score. These findings suggest that timeliness may play a role in the success of TDO for PF pathology. A TDO procedure may be more successful when a patient is younger (<25 years old) before degenerative chondromalacia progresses beyond Grade I.

## 4. Discussion

This review demonstrates several salient trends among studies that warrant further investigation with more rigorous, comparative research methods: (1) TDO results in significantly improved pain and PROM ratings in patients with PF pain and/or instability; (2) the likelihood of complication, including recurrent patella subluxation after TDO, is low but this may increase with age; (3) the successful anatomical correction of TDO may be augmented by concurrent TTT.

Regarding postoperative complications, aside from one study [31], no recurrence of patellar dislocation was reported in subjects whose indication for surgery included PF instability. Stevens et al. used a patient-reported questionnaire to assess long-term outcomes (mean follow-up time of 59 months). The questionnaires revealed that 43% of patients reported subjective instability. Additionally, 70% reported continued knee pain and problems with 22% of them having had subsequent knee surgery after the osteotomy. Despite significant decreases in pain reports (8.6 to 3.3 postoperatively), the subjects in this study appeared to have more difficulty postsurgically than the other included studies. This outlying finding may be due to the study's small sample size and the authors' inclusion of multiple techniques such as lateral retinacular releases, femoral osteotomies, and combined tibial and femoral osteotomies in their subject population.

A meta-analysis of 629 knees that underwent MPFL reconstruction for PF instability found a complication rate of 26.1% [37]. This is remarkably higher than the 12.1% TDO complication rate observed in the present review. This may be due to the fact that MPFL does not address EETT as a potential underlying etiology of PF instability.

Of note, 3 of the 4 included studies that reported osseous malunion postoperatively had a mean subject age of 56 (range 49–62) [14], 27 (13–48) [32], and 34.6 (19–57) [18], which are all over the threshold of 25 years old as defined by Manilov et al. for worsened prognosis after TDO. This, in combination with Manilov et al.'s findings, emphasizes that age, and therefore, the degree of chondral degeneration of the PF joint plays a role in the success of TDO in this setting. Prior studies have found that the mean age of patellar dislocation is 14–18 years, and the mean age of MPFL reconstruction is 23.5 years [1, 38]. Therefore, the ideal patient to undergo surgical correction of PF instability may be young (<25 years old). According to the findings of this review, TDO is no exception to this trend. The reason behind this finding may be due to the level of chronicity of injury and, therefore, the degree of PF chondromalacia [30]. Additionally, increased bone age and poor bone quality have long been associated with malunion in other orthopedic surgeries [39, 40].

Interestingly, many of the subjects included in this review had previous surgeries that were unsuccessful in improving PF pathology [17–19, 30]. Cameron and Saha demonstrated similarly good outcomes with TDO in patients who had previously undergone TTT for PF instability [9]. This suggests that medialization of the tibial tubercle may not be sufficient in correcting PF instability when EETT is present (>30 deg) [14]. Many surgeons elect to perform a combined TTT/TDO procedure which, according to the current review, may provide additional anatomical correction compared to TDO without TTT.

Tibial tuberosity-trochlear groove distance is an established measurement that, when increased, has been associated with recurrent patellar dislocations [41]. However, Tensho et al. found that TTTG does not correlate with tibial tubercle lateralization, a finding that is often considered an indication of TTT [41]. Since TTTG distance does not depend on the lateralization of the tibial tubercle, a TTT (medialization of the tibial tubercle) may not be the best surgical option to correct increased TTTG due to EETT. Symptomatic excessive external tibial torsion may be the bigger contributor to increased TTTG in certain cases, owing to the increase in recurrent subluxation in these patients [42]. Therefore, a TDO may be necessary for the surgical correction of recurrent patellar instability with increased TTTG due to EETT.

Two studies demonstrated that the degree of preoperative femoral anteversion does not influence outcomes in an isolated TDO [19, 30]. In one study that included subjects whose femoral anteversion was corrected with a femoral derotation (in addition to TDO), outcomes and patient satisfaction were both less favorable (56% of patients had recurrent patellar instability at final follow-up) [31]. These findings suggest that a TDO alone may be sufficient for surgical correction of PF instability due to EETT, regardless of the degree of femoral anteversion.

The lack of large, comparative studies on this topic limits this review. Currently, the body of literature on this topic includes almost exclusively case series. Additionally, the heterogeneity among studies, particularly the potentially confounding inclusion of surgical techniques beside TDO, limits this review. However, since the TDO is often used in combination with other techniques, such as TTT, we believe that the inclusion of such articles improves the generalizability of the present review's findings.

## 5. Conclusion

Anatomical correction, PROMs, and pain all appear to improve with tibial derotational osteotomies when performed as indicated for EETT with patellar instability. The outcomes of TDO appear to be negatively associated with advanced age, and patients less than 25 years old appear to have the most favorable outcomes. Future comparative studies should compare TTT to combined TTT/TDO and isolated TDO in order to better inform surgical technique choice in this patient population.

## Figures and Tables

**Table 1:** Search strategy.

1	exp rotation/or (derotation or derotational or rotational or rotation).mp
2	exp tibia/or tibia.mp. or tibial.mp
3	(torsion or torsional).mp
4	1 AND 2 AND 3

**Table 2:** Summary of study characteristics and salient findings.

Author	Procedure (s) performed	Subjects (knees)	Follow-up	Salient findings
Drexler, et al. (2013)	TKA + TDO + TTT^*∗*^	10 (12)	7 years (average)	Anatomical correction
				(i) Average rotational correction of tibial torsion: 30 degrees (range, 20–35)
				Patient reported outcome measures (preop vs postop)
				(i) KSS^*∗*^ part 1 improved from 30.2 ± 11.51 to 86.8 ± 11.75 (*p* < 0.0001); part 2 improved from 28 ± 17.8 to 75.8 ± 11.75 (*p* < 0.0001)
				(ii) SF-12^*∗*^ physical score improved from 31 ± 3.68 to 51.2 ± 8.8 (*p* < 0.0001); mental score improved from 23.8 ± 5.34 to 55 ± 8.6
				(iii) WOMAC^*∗*^ total score improved from 12.74 ± 7.63 to 84 ± 14.95 (*p* < 0.0001)
				Pain
				(i) Visual analogue scale pain ratings improved from 8.5 ± 0.9 to 1.3 ± 1.4 on average
				Patient satisfaction
				(i) 9/10 subjects stated they were “satisfied” with the procedure
				Complications: 5 subjects (5 knees)
				(i) Persistent anterior knee pain (2)
				(ii) Subsequent patellar resurfacing procedure (1)
				(iii) Tibial pain secondary to buttress plate and screws (2)
				(iv) Postoperative stiffness with flexion to only 70 degrees (1)

Dickschas, et al. (2017)	TDO^*∗*^	42 (49)	42 months (range, 6–131)	Anatomical correction
				(i) External tibial torsion was corrected on average by 10.8 deg (SD 3.01; range 5–18)
				Patient-reported outcome measures
				(i) Tegner activity score: increased 0.4 points on average (*p*=0.014)
				(ii) Lysholsm score: increased 26 points on average (*p*=0.001)
				(iii) Japanese knee society score: increased 18 points on average (*p*=0.001)
				Pain and symptoms
				(i) Visual analogue scale pain ratings were reduced by 3.4 points on average (*p*=0.001)
				(ii) Zero subjects reported redislocation of the joint during the follow-up period
				Complications: 4
				(i) No bone healing detectable on X-ray at 4 months (1)
				(ii) Painful fibular pseudarthrosis (1)
				(iii) Compartment syndrome (1)
				(iv) Peroneal nerve deficiency without compartment syndrome (1)

Drexler, et al. (2014)	TDO + TTT^*∗*^	12 (15)	84 months (range, 15–156)	Anatomical correction
				(i) Median preop EETT^*∗∗*^ = 62 degrees (range, 55–70)
				(ii) Median correction = 36 degrees (range, 30–45 degrees)
				Patient reported outcome measures
				(i) KSS^*∗*^ part 1 improved from 37 ± 14 to 89 ± 11 points; part 2 improved from 25 ± 26 to 85 ± 14 points (*p*=0.002)
				(ii) Kujala patellofemoral score improved from 31.3 ± 15.1 to 86.8 ± 12.5 (*p*=0.002)
				(iii) WOMAC^*∗*^ total improved from 27.3 ±1 8.3 to 87.7 ± 10.3 (*p*=0.003)
				(iv) SF-12 physical improved from 32.2 ± 5.7 to 50.4 ± 7.9 (*p*=0.002)
				(v) SF-12 mental improved from 23.8 ± 5.5 to 57 ± 5.6 (*p*=0.002)
				Complications: 2
				(i) Nonunion of the tibial osteotomy (2)

Stevens, et al. (2014)	TDO + femoral osteotomy	16 (23)	59 months (range, 11–145)	Pain
				(i) Improvement in visual analogue scale pain ratings from a mean of 8.6 to 3.3 (*p* < 0.0001)
				Patient-reported questionnaire
				(i) Continued knee instability in 10/23 knees
				(ii) 13/23 knees were “trusted” by patients
				(iii) “Activity levels” improved in 15/23 knees, remained the same in 3 knees, and decreased in 5 knees
				(iv) 2/23 subjects required additional surgery: bilateral guided growth (1) and arthroscopic debridement and microfracture (1)
				Complications: 2
				(i) Femoral nonunion (1)
				(ii) Peroneal nerve injury from loose proximal tibial interlocking screw (1)

Fouilleron, et al. (2010)	TDO	29 (36)	4.7 years	Anatomical correction
				(i) Mean derotation was 25 degrees
				Patient satisfaction
				(i) 27/29 patients were “satisfied” or “very satisfied” with the procedure (94%)
				Patient-reported outcome measures
				(i) Lille score increased from 54.8 ± 16.9 to 85.2 ± 14.2
				(ii) IKS^*∗*^ knee score increased from 56 ± 14.8 to 94 ± 12.1; IKS function score increased from 71 ± 18.4 to 96 ± 11.9 (*p* < 0.05)
				Complications: 4
				(i) Stiffness requiring knee manipulation under general anesthesia (2)
				(ii) Subsequent surgical fibular fibrous arch release (1)
				(iii) Regressive palsy of the common fibular nerve (1)

Manilov, et al. (2020)	TDO	54 (60)	66 months	Patient-reported outcome measures
				(i) Kujala score improved from 47.5 to 93 (*p* < 0.05) on average
				(ii) Fulkerson score improved from 40.6 to 91.6 on average (*p* < 0.05)
				(iii) Kujala subscores for pain improved from 8.6 to 30.4, instability improved from 6.4 to 17.9, and ability to climb stairs increased from 6.9 to 17.9 (all *p* < 0.0001)
				Complications: 5
				(i) Intraoperative proximal tibial fracture distal to the osteotomy (1)
				(ii) Neuroproxia of the peroneal nerve (1)
				(iii) Permanent palsy of EHL (1)
				(iv) Subsequent arthroscopic lysis of adhesions due to postoperative stiffness (2)
				(v) ^*∗*^11 subjects required hardware removal within the first postoperative year due to local pain

Leonardi, et al. (2014)	Unilateral femoral osteotomy (4) Unilateral tibial osteotomy (2) Combined bilateral tibial/femoral osteotomis (3)	9 (20)	16 years	Anatomical correction
				(i) Average preop tibial external torsion = 47 degrees (range, 42–54)
				(ii) Average postop tibial external torsion = 26.3 degrees (range, 30–32)
				Patient-reported symptoms
				(i) At final follow-up, all patients reported “marked relief of symptoms”

^*∗*^TKA = total knee arthroplasty; TDO = tibial derotational osteotomy; TTT = tibial tuberosity transfer; KSS = knee society score; WOMAC = Western Ontario and McMaster University Osteoarthritis Index; SF-12 = short-form 12; IKS = International knee society, ^*∗∗*^EETT = excessive external tibial torsion.

**Table 3:** Mean anatomical correction of each procedure or combination of procedures in the included studies. These findings were confirmed by XR or CT.

Procedure (s) performed	Mean anatomical correction
TDO^*∗*^ (*n* = 147) [19, 30, 32]	19.9 degrees (range, 5–45)
TDO + femoral derotational osteotomy (*n* = 11) [29, 31]	20.8 degrees (range, 5–45)
TDO + TTT^*∗*^ (*n* = 12) [18]	34 degrees (range, 20–45)
TDO + TTT + TKA^*∗*^ (*n* = 10) [17]	30 degrees (range, 20–35)

^*∗*^TDO = tibial derotational osteotomy; TTT = Tibial tuberosity transfer; TKA = total knee arthroplasty.

**Table 4:** Average improvement of patient-reported outcome measures compared to preoperative values.

Patient-reportedoutcome measure	Postoperative improvement at follow-up (average among studies)	Exceeds minimal clinically important difference?
Visual analogue scale (VAS) pain ratings	4.73 points decrease [17, 18, 31, 32]	Yes (MCID = 3.2) [34]
Kujala patellofemoral score	54.2 points increase [18, 30]	Yes (MCID = 13.5) [35]
Knee society score	Part 1: 54.3 points increase part 2: 53.9 points increase [17, 18]	Yes (MCID = 8.6 for cumulative score) [36]
Short form-12	Physical: 19.2 points increase mental: 32.2 points increase [17, 18]	Yes (MCID = 7.2 and 6.3 for physical and mental sections, respectively) [36]

## Data Availability

The data extracted from the included articles can be found in Tables 2–4.
